# Investigating Cu-Site Doped Cu–Sb–S Nanoparticles Using Photoelectron and Electron Paramagnetic Resonance Spectroscopy

**DOI:** 10.1021/acs.jpcc.4c02602

**Published:** 2024-08-08

**Authors:** Jacob E. Daniel, S. Ivan Weaver, Brad R. Matthias, River Golden, Gavin M. George, Christian Kerpal, Carrie L. Donley, Lauren E. Jarocha, Mary E. Anderson

**Affiliations:** † Department of Chemistry, Furman University, Greenville, South Carolina 29613, United States; ‡ Chapel Hill Analytical and Nanofabrication Lab, Department of Chemistry, University of North Carolina, Chapel Hill, North Carolina 27599, United States; § Department of Physics and Astronomy, UNC Asheville, Asheville, North Carolina 28804, United States

## Abstract

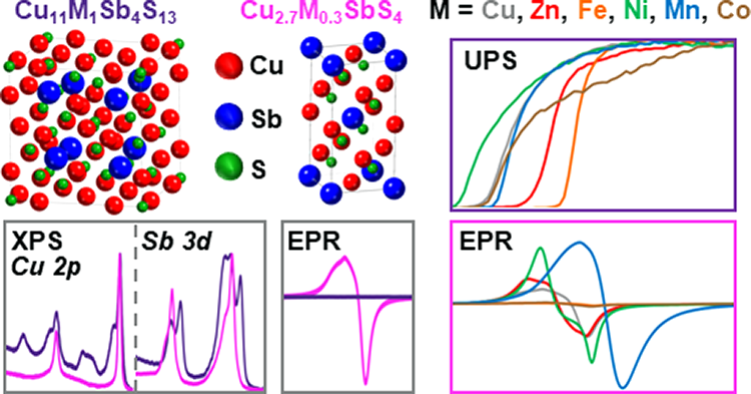

Tetrahedrite (Cu_12_Sb_4_S_13_) and famatinite (Cu_3_SbS_4_) are good candidates for green energy applications because they possess promising thermoelectric and photovoltaic properties as well as contain earth-abundant and nontoxic constituents. Herein, X-ray photoelectron spectroscopy (XPS), ultraviolet photoelectron spectroscopy (UPS), and electron paramagnetic resonance spectroscopy (EPR) methods examined inherent electronic properties and interatomic magnetic interactions of Cu-site doped tetrahedrite and famatinite nanomaterials. An energy-efficient modified polyol method was utilized for the synthesis of tetrahedrite and famatinite nanoparticles doped on the Cu-site with Zn, Fe, Ni, Mn, and Co. This is the first parallel study of tetrahedrite and famatinite nanomaterials with XPS, UPS, and EPR methods alongside a systematic analysis of dopant-dependent effects on the electronic structure and magnetic interactions for each material. XPS showed that the Cu and Sb species in tetrahedrite and famatinite possess different oxidation states, while UPS characterization reveals larger dopant-dependent shifts in the work function for tetrahedrite nanoparticles (4.21 to 4.79 eV) than for famatinite nanoparticles (4.57 to 4.77 eV). Finally, all famatinite nanoparticles display an EPR signal, indicating trace amounts of paramagnetic Cu(II) present below the detection limit of XPS. For tetrahedrite, EPR signatures were observed only for the Zn-doped and Mn-doped nanoparticles, suggesting signal broadening from Cu–Cu spin exchange or spin–lattice relaxation. This study demonstrates the complementary nature of XPS and EPR techniques for studying the oxidation states of metals in solid-state nanomaterials. Comparing the electronic and magnetic properties of tetrahedrite and famatinite while studying the impact of dopant incorporation will guide future endeavors in designing sustainable, high-performance materials for renewable energy applications.

## 1 Introduction

Nanoparticles of the ternary Cu–Sb–S family, specifically tetrahedrite (Cu_12_Sb_4_S_13_) and famatinite (Cu_3_SbS_4_), are composed of earth-abundant, nontoxic constituents, and possess promising physical properties for thermoelectric and photovoltaic applications.^1−6^ Thermoelectric waste heat recycling and photovoltaic electricity generation technologies may be central to transitioning away from the global use of greenhouse gas emitting fossil fuels for energy generation.^7−10^ High-performing semiconducting nanocrystals such as Bi_2_Te_3_ and PbTe (thermoelectrics) or CdTe and InGaAs (photovoltaics) are commercially available; however, adoption of these materials is limited by reliance on expensive rare-earth and sometimes toxic constituent elements.^5,6^ As a result, emphasis has been placed on developing and optimizing alternative thermoelectric and photovoltaic semiconducting nanomaterials, such as tetrahedrite and famatinite.^1−4^ Dopant incorporation has been utilized to enhance thermoelectric performance, increase thermal stability, and modify optical properties of metal chalcogenide materials.^11−14^ The addition of dopants is known to modify the density of states around the Fermi level in semiconducting materials, which allows doping to impact material properties like the Seebeck coefficient, electrical conductivity, and band gap.^12^ X-ray photoelectron spectroscopy (XPS), ultraviolet photoelectron spectroscopy (UPS), and electron paramagnetic resonance (EPR) spectroscopy are analytical characterization methods that, in tandem, can examine oxidation states, electron binding energies, ion coordination environments, material work functions, and identity of paramagnetic species. Herein, these methods were used to study the electronic structure and magnetic interactions of tetrahedrite and famatinite nanoparticles, as well as to investigate the impact of dopant incorporation for each material.

**Figure 1:**
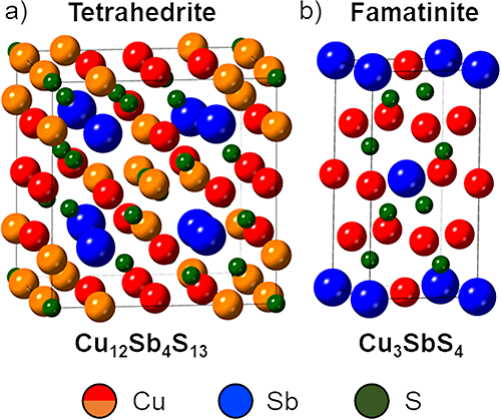
Unit cells of (a) tetrahedrite^15^ (Cu_12_Sb_4_S_13_) and (b) famatinite^16^ (Cu_3_SbS_4_). In the tetrahedrite unit cell (a), the tetrahedral Cu site is labeled in red and the trigonal Cu site in orange. In the famatinite unit cell (b), all Cu atoms are colored red.

The crystal structures of tetrahedrite and famatinite compounds are shown in Figure 1a,b. The cubic tetrahedrite unit cell is large (52 atoms), while famatinite has a smaller (16 atom) tetrahedral unit cell.^4,17−19^ All Cu atoms in famatinite are tetrahedrally coordinated to four sulfur atoms,^18^ whereas Cu atoms within the tetrahedrite unit cell exist in both tetrahedral and trigonal planar arrangements with neighboring sulfur atoms.^4,17^ Furthermore, vacancy sites present within the unit cell of natural and synthetic tetrahedrites allow for the incorporation of extra Cu or dopant atoms, resulting in a compositional range of Cu_12–14.5_Sb_4–4.5_S_13_.^20^ Cu-poor tetrahedrite (Cu_12_Sb_4_S_13_) contains two Cu(II) species and ten Cu(I) species, while Cu-rich tetrahedrite with occupied vacancy sites (Cu_14_Sb_4_S_13_) contains exclusively Cu(I) species. No such vacancy sites are present in famatinite; therefore, all Cu species occupy the Cu(I) oxidation state. These differences in the crystal structures impact the physical properties of the materials. Tetrahedrite has a uniquely low thermal conductivity, with one study finding thermal conductivity between 0.5 and 1.5 W·m^–1^·K^–1^ from 300 to 800 K for both synthetic tetrahedrite (Cu_12–*x*_Zn_*x*_Sb_4_S_13_, *x* = 0, 0.5, 1, 1.5) and natural tetrahedrite mineral mixed with undoped synthetic tetrahedrite.^21^ This favorable thermal conductivity is in part engendered by an anharmonic “rattling” effect due to interatomic interactions between Sb lone pairs and a trigonally coordinated Cu atom located between two Sb atoms.^4^ Famatinite, lacking this anharmonic rattling, consequentially possesses a higher thermal conductivity (1–4 W·m^–1^·K^–1^) than tetrahedrite.^22^ Both materials exhibit promising but distinct optical properties that render them viable for photovoltaic applications. Tetrahedrite has an absorption coefficient on the order of 10^–4^ cm^–1^ and a band gap ranging from 1.5–2.0 eV, while famatinite possesses a slightly superior absorption coefficient on the order or 10^–5^ cm^–1^ and a lower energy band gap of between 0.5–1.2 eV.^3,17−19^

The tetrahedrite and famatinite nanoparticles studied herein are synthesized by a facile, bottom-up, solution-phase modified polyol method. This modified polyol method was developed to provide a rapid, energy-efficient, and easily scalable process that has produced ligand- and surfactant-free semiconducting nanoparticles, such as ternary Bi–Sb–Te and Cu–Sb–S compounds.^23−27^ Additionally, the modified polyol process allows for tailored dopant incorporation in both tetrahedrite and famatinite compounds.^24−27^ Previous studies have examined the growth mechanism, structural properties, thermal stability, and optical behavior of tetrahedrite and famatinite doped on the Cu-site with Zn, Fe, Ni, Mn, and Co.^26,27^ Other solution-phase methods such as hot injection or solvothermal procedures have been used to synthesize nanoscale tetrahedrite and famatinite.^19,28−30^ However, the products of these syntheses are coated with ligands or surfactants that can lower thermoelectric and photovoltaic performance; and oftentimes the incorporation of dopants at tunable levels is difficult.^23−25^ Typically, tetrahedrite and famatinite are synthesized by solid-state methods like melting and annealing or mechanical alloying processes, producing large quantities of bulk material;^21,22,31−36^ but these methods are time-consuming, energy-intensive, and susceptible to the formation of impurity phases.

Alongside more typical thermoelectric and optical characterization, some studies have utilized XPS and UPS methods to experimentally examine the electronic properties of tetrahedrite and famatinite materials.^28,34−42^ XPS and UPS analyzed oxidation states of constituent elements, investigated electron binding energies, derived the material work function, and studied the overall electronic structure of tetrahedrite and famatinite materials.^28,34−42^ One study presented XPS spectra (Cu 2p and S 2p regions) for tetrahedrite and famatinite nanomaterials synthesized by a hot injection process, finding Cu(II) in tetrahedrite and only Cu(0) or Cu(I) species in famatinite; and they obtained work functions of 4.7 and 4.8 eV for tetrahedrite and famatinite, respectively.^28^ However, this study did not synthesize doped tetrahedrite and famatinite material, and thus the impact of doping on electronic structure was not discerned. To the authors’ knowledge, only one study has utilized XPS and UPS methods to characterize and compare the electronic properties of tetrahedrite materials doped with a variety of transition metals.^36^ In the photoelectron characterization of solid-state synthesized tetrahedrite materials, the oxidation states of dopant species were derived from the XPS spectra, and the identity of the Cu site in which substitution occurred was investigated.^36^ However, this study did not characterize samples with a uniform dopant concentration; therefore, a direct comparison regarding the impact of the dopant on physical properties could not be attained. Additionally, to the author’s knowledge, no studies have investigated doped famatinite using XPS and UPS methods. Dopant-dependent changes to the electronic structure of tetrahedrite and famatinite could be reflected in the XPS and UPS spectra of the materials, specifically by observing peak shifts or changes in work function. Analyzing the changes in electronic structure when dopants are systematically incorporated at standard levels may provide insight into how doping impacts other material properties, such as thermal stability or optical absorbance.

Introducing transition-metal dopant species to tetrahedrite and famatinite could result in dopant-dependent changes to magnetic interactions within the material in addition to altering the electronic structure. Steady-state EPR allows for the identification of paramagnetic species in a material and has a high sensitivity. A typical spectrometer operating at X-band has a theoretical detection limit of 10^10^ spins at room temperature.^43,44^ EPR is commonly used to study a wide range of compounds, including organic radicals, transition metal complexes, and solid-state conducting materials like semiconductors or intermetallics.^43−54^ By focusing on unpaired electrons within a material, EPR provides specific information about the local coordination environment of paramagnetic species or spin–spin and spin–lattice interactions, as well as general information about magnetic properties and electronic structure.^44^ EPR studies of multinary copper sulfide materials such as covellites,^47,48^ CZTS kesterite,^49,50^ and natural and synthetic tetrahedrite and famatinite^51−54^ have been performed previously. There are two studies that looked to analyze the magnetic behavior of undoped and Fe-doped tetrahedrite or undoped and Ni-doped famatinite compounds, respectively.^53,54^ However, no studies have investigated and compared the EPR signatures of tetrahedrite and famatinite nanomaterials doped with a variety of transition metals.

In this study, the electronic structure and magnetic interactions within polyol-synthesized tetrahedrite and famatinite nanoparticles were evaluated using XPS, UPS, and EPR methods. XPS characterization of the Cu 2p, Sb 3d, and S 2p regions obtained the oxidation states of constituent atoms in tetrahedrite and famatinite nanomaterials. Analysis of Cu-site doping with transition metals on the electronic structure of tetrahedrite and famatinite nanoparticles was undertaken by searching for dopant-dependent peak shifts in the XPS spectra. UPS studies were conducted in tandem to derive the work functions of undoped and doped tetrahedrite and famatinite nanomaterials. Furthermore, similarities and differences in the electronic structure of polyol-synthesized tetrahedrite and famatinite nanoparticles were evaluated. EPR was used to identify trace paramagnetic species and to provide complementary analysis of dopant-dependent changes to electronic structure of tetrahedrite and famatinite nanomaterials doped on the Cu-site with a series of different transition metals for the purpose of comparison. Prior to XPS, UPS, and EPR characterization, phase purity and elemental composition of tetrahedrite and famatinite samples were analyzed with powder X-ray diffraction, scanning electron microscopy, and energy dispersive X-ray spectroscopy. Understanding the impact of dopants on the electronic structure and magnetic behavior of tetrahedrite and famatinite as well as comprehending the differences between the materials can provide valuable insight into the design of effective thermoelectric and photovoltaic materials.

## 2 Experimental Section

### 2.1 Materials

The following reagents from Sigma-Aldrich Chemical Co. were used as received for the synthesis of tetrahedrite (Cu_12_Sb_4_S_13_) and famatinite (Cu_3_SbS_4_) nanoparticles: antimony(III) acetate, (Sb(C_2_H_3_O_2_)_3_, ≥99.99%), copper(II) acetate monohydrate (Cu(C_2_H_3_O_2_)_2_·H_2_O, ≥98%), sulfur powder (99.98%), zinc(II) acetate (Zn(C_2_H_3_O_2_)_2_, 99.99%), iron(II) acetate (Fe(C_2_H_3_O_2_)_2_, 95%), nickel(II) acetate tetrahydrate (Ni(C_2_H_3_O_2_)_2_·4H_2_O, 98%), manganese(II) acetate (Mn(C_2_H_3_O_2_)_2_, 98%), and cobalt(II) acetate (Co(C_2_H_3_O_2_)_2_, 99.99%). Synthesis was completed using tetraethylene glycol (99%) from Alfa Aesar and ThermoScientific as the solvent, and the addition of sodium borohydride powder (NaBH_4_, ≥98.0%) from Sigma-Aldrich modified the polyol process. Products were isolated with anhydrous ethanol (200 proof, USP grade) from Pharmco-Aaper.

### 2.2 Synthesis of Tetrahedrite and Famatinite Nanoparticles

The modified polyol synthesis of tetrahedrite and famatinite nanoparticles followed procedures published in the literature^23−27^ and is summarized below.

#### 2.2.1 Tetrahedrite

To synthesize ∼0.7 g of undoped tetrahedrite (Cu_12_Sb_4_S_13_) nanoparticles, 5.0 mmol (1.0 g) of Cu(C_2_H_3_O_2_)_2_·H_2_O, 1.7 mmol (0.50 g) of Sb(C_2_H_3_O_2_)_3_, and 5.4 mmol (0.17 g) of sulfur were dissolved in 50 mL tetraethylene glycol in a 250 mL round-bottom flask. For the synthesis of single-doped tetrahedrite (Cu_11_M_1_Sb_4_S_13_, M= Zn, Fe. Ni, Mn, or Co) nanoparticles, the only modifications were that 4.6 mmol (0.92 g) of Cu(C_2_H_3_O_2_)_2_·H_2_O and 0.42 mmol of one of the following were added to the flask: Zn(C_2_H_3_O_2_)_2_ (0.077 g), Fe(C_2_H_3_O_2_)_2_ (0.073 g), Ni(C_2_H_3_O_2_)_2_·4H_2_O (0.10 g), Mn(C_2_H_3_O_2_)_2_ (0.073 g), or Co(C_2_H_3_O_2_)_2_ (0.074 g). In all syntheses, the combination of these reagents resulted in a light blue solution that was sparged with N_2_ gas while stirred constantly for 10 min. Sodium borohydride (NaBH_4_, 26 mmol, 1.0 g) was suspended in 30 mL tetraethylene glycol with the assistance of sonication and added slowly to reagent mixture (1 mL aliquots over the span of ∼5 min), changing the color to dark brown. The solution was heated under N_2_ in a reflux setup to 220 °C and held for 1 h with magnetic stirring. The resulting nanoparticles were washed with ethanol three times by centrifugation for 10 min at 4000 rpm. The final product was dried in a vacuum chamber overnight. Undoped tetrahedrite product was dark brown in color, while single-doped tetrahedrite products were light brown in color except for the Zn-doped product, which was brick red.

#### 2.2.2 Famatinite

For the synthesis of ∼0.4 g undoped famatinite (Cu_3_SbS_4_) nanoparticles, 3.0 mmol (0.60 g) Cu(C_2_H_3_O_2_)_2_·H_2_O, 1.0 mmol (0.30 g) Sb(C_2_H_3_O_2_)_3_, and 4.4 mmol (0.14 g) of sulfur powder were dissolved in 30 mL tetraethylene glycol in a 250 mL round-bottom flask. To synthesize single-doped famatinite (Cu_2.7_M_0.3_SbS_4_, M = Zn, Fe. Ni, Mn, or Co) nanomaterial, the amount of Cu(C_2_H_3_O_2_)_2_·H_2_O was altered to 2.7 mmol (0.54 g) and an additional 0.30 mmol of dopant precursor, such as Zn(C_2_H_3_O_2_)_2_ (0.055 g), Fe(C_2_H_3_O_2_)_2_ (0.052 g), Ni(C_2_H_3_O_2_)_2_·4H_2_O (0.075 g), Mn(C_2_H_3_O_2_)_2_ (0.052 g), or Co(C_2_H_3_O_2_)_2_ (0.053 g), was added. The synthesis of famatinite is similar to that of tetrahedrite described above with the following alterations: 15 mmol (0.60 g) sodium borohydride (NaBH_4_) dispersed in 20 mL tetraethylene glycol was utilized and the reaction was held at 175 °C. All famatinite powders appeared matte black in color.

### 2.3 Characterization Techniques

XRD, SEM, and EDS methods were utilized to study the structure and composition of tetrahedrite and famatinite nanomaterials. Electronic properties were characterized with XPS and UPS, while EPR was used to study paramagnetic species within the sample.

Experimental nanoparticle XRD patterns were obtained with a Rigaku Miniflex II benchtop diffractometer. Patterns were collected with 30 kV and 15 mA Cu Kα radiation over a 2θ range of 10° to 70°. The scan speed was 1° or 3° per minute (depending on what was required to yield a Chi^2^ of >1) and the scan width is 0.03°. The PDXL2 software package was utilized to perform Rietveld refinements of the patterns to derive the lattice constants and crystallite sizes of tetrahedrite and famatinite nanoparticles. PDF Cards #01-071-0270^15^ and #01-074-0555^16^ were used to match the tetrahedrite and famatinite phases, respectively.

Electron microscopy and elemental compositional analysis were performed using a JEOL JSM IT-200LA scanning electron microscope equipped with a JEOL JED-2300 Dry SDD EDS detector. SEM images and EDS data were acquired with an accelerating voltage of 15 keV. Semiquantitative EDS analysis was undertaken at three or more locations throughout the sample to ensure homogeneity. Elemental ratios were derived from calculated averages at three or more locations and include standard deviations. Standard deviations and significant figures are representative of spot-to-spot variation in composition for the multiple regions sampled.

XPS data was collected on a Kratos Supra+ system with a monochromatic Kα X-ray source operated at 150 W. A charge neutralizer was used to prevent charging when necessary and all spectra were corrected to the C 1s peak at 284.6 eV. While some drawbacks for this energy correction method have been noted in the literature,^55,56^ the C 1s energy correction method is the only one available in this case as no other suitable elements are available for reference. Survey and high-resolution scans were acquired at pass energies of 80 and 20 eV, respectively, and the analyzed spot size was 300 × 700 μm. XPS peak fitting was done with the Kratos Escape software. Shirley baselines were used, and Gauss* Lorentz blend = 0.3 peaks were used.

UPS data was collected on the same system using a He I UV source operated at 20 mA with an ionization energy of 21.2 eV. Data was acquired with a pass energy of 5 eV, a 9 V bias was applied to the sample, and the 55 μm aperture was used to prevent saturation of the detector. No charge neutralizer was used for UPS measurements.

Electron paramagnetic resonance (EPR) spectroscopy data was collected on a Bruker Magnettech ESR5000 (X-band) as an average of 4 scans. All experiments were performed with 5–10 mg of sample placed in a quartz capillary with an ID of 0.9 mm. EPR spectra were collected at 1 mW of power and a field sweep time of 167 s. Modulation amplitude was kept at or below 0.3 mT. Measurements were taken with a center field of 300 mT and an average microwave frequency of 9.45 GHz.

## 3 Results and Discussion

A systematic study of the structural, electronic, and magnetic properties of Cu-site transition metal doped tetrahedrite and famatinite nanoparticles synthesized by a facile and versatile polyol synthetic method is presented herein. Tetrahedrite (Cu_11_MSb_4_S_13_, M = Cu, Zn, Fe, Ni, Mn, or Co) and famatinite (Cu_2.7_M_0.3_SbS_4_, M = Cu, Zn, Fe, Ni, Mn, or Co) nanoparticles were first characterized with XRD, SEM, and EDS to confirm phase purity and ascertain elemental composition. XPS and UPS characterization of tetrahedrite and famatinite was performed to determine oxidation states of constituent atoms, derive the work function of tetrahedrite and famatinite, and compare material electronic properties. Dopants incorporated into the lattice are investigated to determine dopant-dependent changes to the electronic structure of tetrahedrite and famatinite nanoparticles, which are indicated by shifts in the XPS and UPS spectra to lower or higher binding energies. EPR spectroscopy is employed to detect paramagnetic species in undoped and doped tetrahedrite and famatinite nanoparticles. The high sensitivity of EPR allows for the detection of trace species and dopants that are not seen with XPS analysis, either because the concentration of those paramagnetic species are below the detection limit of XPS or because they are located in the bulk rather than surface of the particles. Throughout this manuscript, the same batch of samples was studied by all characterization methods aforementioned.

### 3.1 Synthetic Characterization

**Figure 2:**
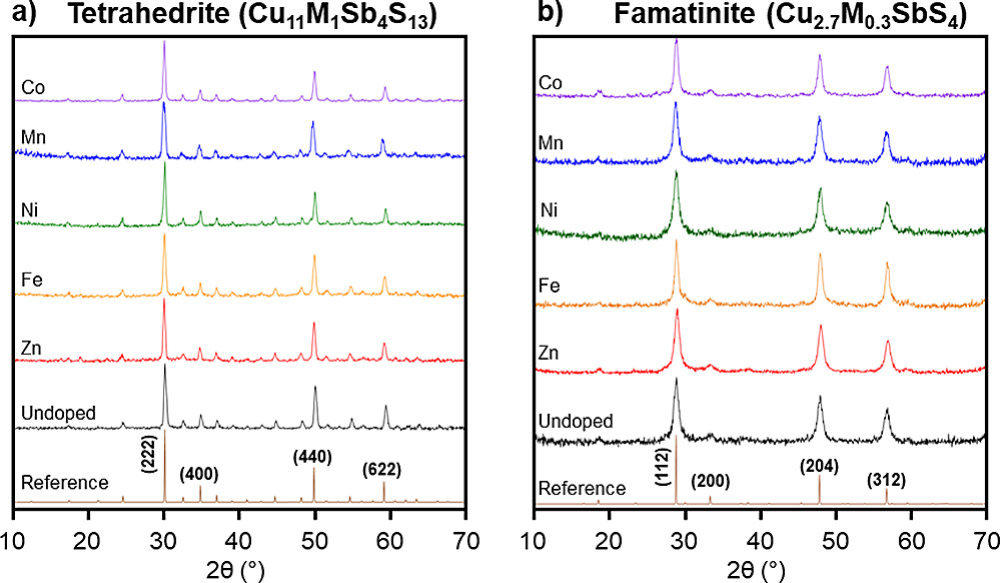
(a) Powder XRD patterns for undoped (Cu_12_Sb_4_S_13_) and doped (Cu_11_M_1_Sb_4_S_13_, M *=* Zn, Fe, Ni, Mn, or Co) tetrahedrite nanoparticles with associated reference pattern^15^ and (b) powder XRD patterns for undoped (Cu_3_SbS_4_) and doped (Cu_2.7_M_0.3_SbS_4_, M = Zn, Fe, Ni, Mn, and Co) famatinite nanoparticles with associated reference pattern.^16^ Patterns are labeled according to the identity of the dopant (M) and the four most intense peaks are indexed on each reference.

Experimental powder X-ray diffraction patterns for tetrahedrite and famatinite nanoparticles are displayed in Figure 2a,b, respectively. For all tetrahedrite and famatinite samples, the diffraction patterns consistently aligned in position and relative intensity to the provided references^15,16^ and a lack of extraneous peaks demonstrated that no crystalline impurities were present. Rietveld refinement calculations were performed on the XRD patterns of all tetrahedrite and famatinite nanoparticles to extract lattice parameters and crystallite sizes for each sample, shown in Tables S1 and S2, respectively. Shifts in the lattice parameters indicative of dopant incorporation are consistent with the literature.^26,27,32,36^ Rietveld refinement calculations determined that the tetrahedrite nanoparticles on average possessed grain sizes (190 ± 30 Å), three times larger than the grain sizes of the famatinite nanoparticles (70 ± 20 Å).

Tetrahedrite nanoparticles were synthesized phase-pure with acceptable elemental compositions and homogeneously distributed elements. Elemental analysis of tetrahedrite and famatinite nanoparticles completed using EDS methods is presented in Table 1. The ratios reported are averages of three or more locations from each sample. The undoped tetrahedrite nanoparticles possessed an elemental composition of Cu_13.7±0.3_Sb_4.40±0.07_S_13.0±0.2_, which is within the accepted compositional range for natural and synthetic tetrahedrite materials (Cu_12–14.5_Sb_4–4.5_S_13_).^20,24,25,27^ The doped tetrahedrite nanoparticles (target composition Cu_11_MSb_4_S_13_, M = Zn, Fe, Ni, Mn, or Co) had an average composition of Cu+Dopant_13.0±0.3_Sb_4.39±0.05_S_13.0±0.2_. All tetrahedrite samples exhibited Cu or Cu+Dopant and Sb enrichment relative to the target ratios of Cu = 12 or Cu+Dopant = 12 and Sb = 4. The doped tetrahedrite nanoparticles contained significantly less Cu or Cu+Dopant enrichment (9% ± 2%) than the undoped nanoparticles (14%). Both undoped and doped tetrahedrite nanomaterials show similar levels of Sb enrichment (10% ± 4%) relative to the target amount of Sb. Low absolute errors with an average relative standard deviation of 5.7% for all tetrahedrite elemental ratios revealed that tetrahedrite samples are homogeneous with no major amorphous impurities present.

**Table 1:** Elemental Composition of Cu–Sb–S Nanoparticles Determined by EDS^a^

tetrahedrite target	Cu ratio	dopant ratio	Sb ratio	S ratio
Cu_11_CoSb_4_S_13_	11.5 ± 0.3	1.02 ± 0.09	4.42 ± 0.04	13.0 ± 0.4
Cu_11_MnSb_4_S_13_	11.9 ± 0.2	1.25 ± 0.03	4.39 ± 0.02	13.0 ± 0.2
Cu_11_NiSb_4_S_13_	12.3 ± 0.2	1.2 ± 0.1	4.67 ± 0.05	13.00 ± 0.06
Cu_11_FeSb_4_S_13_	12.1 ± 0.1	0.97 ± 0.07	4.2 ± 0.1	13.00 ± 0.03
Cu_11_ZnSb_4_S_13_	11.8 ± 0.4	1.2 ± 0.1	4.25 ± 0.03	13.0 ± 0.5
Cu_12_Sb_4_S_13_	13.7 ± 0.3		4.40 ± 0.07	13.0 ± 0.2

famatinite target	Cu ratio	dopant ratio	Sb ratio	S ratio
Cu_2.7_Co_0.3_SbS_4_	2.58 ± 0.04	0.31 ± 0.03	1.072 ± 0.009	4.00 ± 0.05
Cu_2.7_Mn_0.3_SbS_4_	3.2 ± 0.2	0.24 ± 0.02	1.08 ± 0.05	4.0 ± 0.1
Cu_2.7_Ni_0.3_SbS_4_	2.88 ± 0.04	0.25 ± 0.03	1.08 ± 0.01	4.00 ± 0.03
Cu_2.7_Fe_0.3_SbS_4_	2.6 ± 0.1	0.287 ± 0.006	1.02 ± 0.02	4.0 ± 0.1
Cu_2.7_Zn_0.3_SbS_4_	2.90 ± 0.03	0.31 ± 0.01	1.02 ± 0.01	4.00 ± 0.04
Cu_3_SbS_4_	3.08 ± 0.04		1.120 ± 0.005	4.00 ± 0.04

a EDS data collected for an average of three or more spots per sample. All atomic ratios are relative to sulfur, which is normalized to 13 for tetrahedrite and 4 for famatinite.

Elemental analysis confirms that phase-pure famatinite nanoparticles with elemental compositions close to the target ratios (Cu_3_SbS_4_ for undoped material and Cu_2.7_M_0.3_SbS_4_ (M = Zn, Fe, Ni, Mn, or Co) for doped products were produced with homogeneous elemental distributions. Undoped famatinite nanoparticles exhibited an elemental composition of Cu_3.08±0.04_Sb_1.120±0.005_S_4.00±0.04_, revealing Cu and Sb enrichment relative to target values of approximately 3 and 12%, respectively. Unlike doped tetrahedrite samples, the doped famatinite nanoparticles did not consistently display Cu+Dopant enrichment; instead, Cu+Dopant ratios ranged from 2.89 ± 0.05 to 3.4 ± 0.2. Additionally, doped famatinite nanomaterials exhibited similar levels of Sb enrichment (6% ± 4%) to the tetrahedrite samples (10% ± 4%). Overall, the doped famatinite nanoparticles possessed an average composition of Cu+Dopant_3.1±0.2_Sb_1.07±0.03_S_4.00±0.06_, with low absolute and relative standard deviations. The average relative standard deviation for all famatinite elemental ratios is 3%, which suggests that the polyol-synthesized famatinite nanoparticles contained minimal amorphous impurities.

### 3.2 Photoelectron Spectroscopy

Herein, a novel investigation for a broad-range of nanoscale doped tetrahedrite and famatinite compounds using XPS and UPS methods is presented. Nanoparticles were analyzed in the Cu 2p, Sb 3d, and S 2p regions of the XPS spectrum. XPS characterization was utilized to identify the oxidation state for constituent elements in both tetrahedrite and famatinite nanoparticles. Additionally, shifts in the XPS spectra were used to investigate the impact of doping on the electronic structure of tetrahedrite and famatinite nanomaterials. UPS spectra were used to calculate the work function for tetrahedrite and famatinite nanoparticle samples. Finally, similarities and differences observed in the XPS and UPS spectra of tetrahedrite and famatinite materials are discussed.

**Figure 3:**
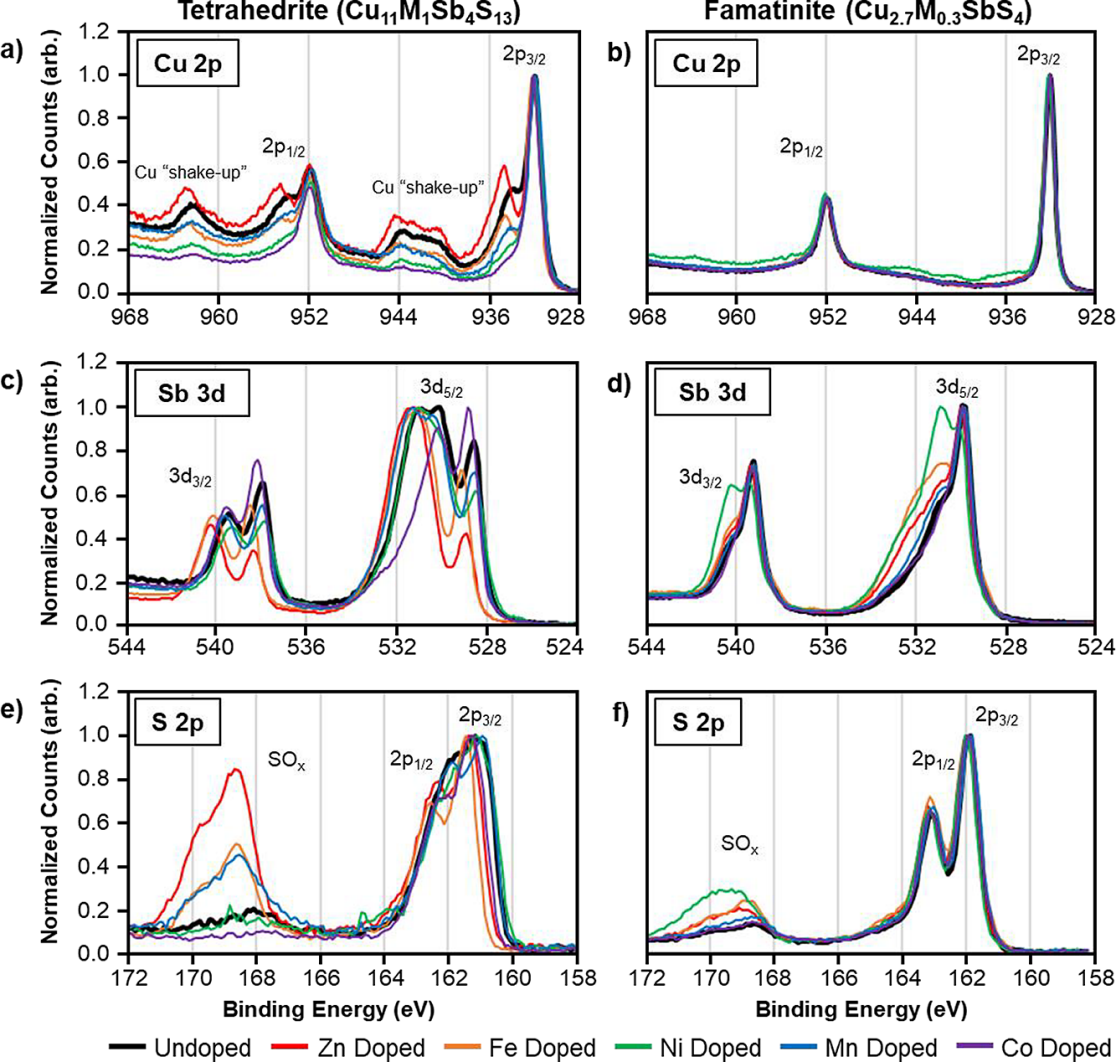
XPS spectra of the Cu 2p, Sb 3d, and S 2p regions for tetrahedrite (Cu_11_M_1_Sb_4_S_13,_ M = Zn, Fe, Ni, Mn, Co) nanoparticles (a, c, e) and famatinite (Cu_2.7_M_0.3_SbS_4_, M = Zn, Fe, Ni, Mn, Co) nanoparticles (b, d, f). The legend identifies samples based on the identity of the dopant species (M), i.e., “Zn Doped” refers to the Cu_11_ZnSb_4_S_13_ tetrahedrite sample or the Cu_2.7_Zn_0.3_SbS_4_ famatinite sample. The undoped samples (black lines) are double the size of other lines for reference purposes.

Figure 3a,b display the Cu 2p region of the XPS spectra for tetrahedrite and famatinite nanoparticles, respectively. In most tetrahedrite samples (Figure 3a), six distinct peaks were observed, including two Cu 2p_3/2_ peaks (932 and 935 eV), two Cu 2p_1/2_ peaks (952 and 955 eV), and two broad satellite features referred to as Cu shake-up peaks (940–944 and 962–964 eV). The lower binding energy 2p_1/2_ and 2p_3/2_ peaks can be attributed to the presence of either Cu(0) or Cu(I), while the higher binding energy 2p_3/2_ and 2p_1/2_ peaks (or shoulders) as well as the Cu shake-up satellite features indicate the presence of Cu(II).^28,34−36,54,57,58^ The relative intensities of the Cu(II) features vary from sample to sample, indicating varying amounts of Cu(II) species within the lattice due to compositional variations. The Zn-doped sample had the most Cu(II), while the Co-doped sample had the least Cu(II). However, CuO species can also contribute to these signals, thereby suggesting tetrahedrite nanoparticles could contain small quantities of amorphous CuO, likely on the nanoparticle surface.^57−59^ The Cu 2p_1/2_ and Cu 2p_3/2_ peaks for famatinite (Figure 3b) are found at 932 and 952 eV, respectively. The famatinite Cu 2p peaks were at the same binding energy as the lower binding energy 2p_1/2_ and 2p_3/2_ peaks in the tetrahedrite spectra. The additional 2p_3/2_ and 2p_1/2_ peaks and the Cu shake-up satellite features seen in tetrahedrite were absent in the famatinite spectra, suggesting that famatinite nanoparticles contained only Cu(0) or Cu(I) species.^28,40−42,57,58^ To further determine if the Cu 2p_3/2_ peak at 932 eV is the result of Cu(0) or Cu(I) species, the Cu Auger regions of the XPS spectra for both compounds were analyzed and are shown in Figure S1. The Cu Auger signal for tetrahedrite (Figure S1a) was broader than for famatinite (Figure S1b), and was shifted to lower binding energies.^58^ This is consistent with the presence of Cu(II) in tetrahedrite; however, the broadness makes the identification of Cu(0) or Cu(I) inconclusive.^58^ Corresponding analysis of the Cu Auger region of the famatinite nanoparticle spectra indicated that all Cu species in the famatinite are Cu(I). These findings for the oxidation states of Cu in tetrahedrite and famatinite materials are consistent with the literature.^28,34−42^ In general, the inclusion of dopants in both tetrahedrite and famatinite did not appear to remarkably impact the binding energies of the Cu species, as no significant peak shifts were observed in the XPS spectra.

The Sb 3d region of the tetrahedrite and famatinite XPS spectra are displayed in Figure 3c,d, respectively. Analysis of the Sb 3d region is more complicated because of an overlap between the Sb 3d_5/2_ peak (527–533 eV) and the O 1s peak (530–535 eV), and as a result, discussion will focus on the Sb 3d_3/2_ peak. Representative peak fits for the Sb 3d region for the tetrahedrite and famatinite samples are shown in Figure S2a,b, respectively. As with the Cu 2p data, there were differences between Sb 3d peaks for tetrahedrite and famatinite. The Sb 3d_3/2_ signal for tetrahedrite (Figure 3c) consists of two peaks, with the binding energies of the lower energy peak ranging from 537.7–538.6 eV and the binding energies of the higher energy peak ranging from 539.6–540.2 eV, varying from sample to sample. The lower binding energy Sb_3/2_ peak is consistent with Sb(III)-S interactions.^57,58^ The higher binding energy 3d_3/2_ peak is separated from the lower energy peak by ∼2 eV, suggesting that oxidized Sb compounds were present likely on the surface of the tetrahedrite nanoparticles.^60^ For all famatinite nanoparticles (except the Ni-doped sample), two more closely spaced peaks were observed; a main peak present at ∼539.5 eV and a shoulder at ∼540 eV. The lower binding energy 3d_3/2_ peak was shifted to a higher binding energy relative to the 3d_3/2_ peak in the tetrahedrite samples, revealing that Sb species in famatinite occupy the Sb(V) oxidation state as opposed to Sb(III) in tetrahedrite.^38,57,58^ The binding energy of the 3d_3/2_ shoulder in the famatinite spectra is consistent with the presence of oxidized Sb species, but the relative intensity of this “shoulder” peak is significantly lower than what was observed in the tetrahedrite spectra. Dopant-dependent shifts in the XPS signals were observed in the Zn-doped and Fe-doped tetrahedrite nanoparticles, with those spectra of the Zn-doped and Fe-doped tetrahedrite nanoparticles shifted to ∼0.5 eV higher binding energies relative to the undoped tetrahedrite nanoparticles. However, no observable shifts were detected in any of the famatinite samples. While minor shifts in binding energy may occur due to artifacts in the C 1s binding energy correction,^55,56^ the famatinite samples having significantly less variation than the tetrahedrite shows that the shifts observed for the tetrahedrite samples are likely not due to these possible artifacts.

The S 2p region of the XPS spectra for tetrahedrite and famatinite nanomaterials are shown in Figure 3e,f. For tetrahedrite samples, S 2p_3/2_ peaks ranged from 161.0–161.5 eV, while for famatinite the 2p_3/2_ peaks were closely grouped at ∼162 eV. As seen in the Sb 3d region, the famatinite S 2p_3/2_ peaks were shifted to higher binding energies relative to the tetrahedrite peaks. The binding energies of the famatinite 2p_3/2_ peaks were notably higher than the range attributed to Cu_2–*x*_S and Sb_*x*_S_*y*_ interactions in the literature.^57,58^ However, these higher binding energy S 2p peaks in famatinite are consistent with the XPS spectra of enargite (Cu_3_AsS_4_) material, a Cu–As–S analogue of famatinite.^61^ Additionally, a study examining the S 2p spectra of hot-injection synthesized tetrahedrite and famatinite nanoparticles also showed that the 2p_3/2_ peak of famatinite nanoparticles were shifted to higher binding energies relative to tetrahedrite nanoparticles.^28^ Most tetrahedrite and famatinite spectra also contain a broad feature from 168–172 eV that revealed the existence of surface SO_*x*_ compounds.^28,57,58^ The Zn-, Fe-, and Co-doped tetrahedrite nanoparticles exhibited dopant-dependent spectral shifts to higher binding energies in the S 2p region, similar to the shifts observed for the Zn- and Fe-doped tetrahedrite nanomaterials in the Sb 3d region. Once again, no dopant-dependent shifts were observed for the spectra of the famatinite nanoparticles.

Analyzing the XPS spectra of tetrahedrite and famatinite reveals several differences between the two materials, with key distinctions being the oxidation states of constituent elements, dopant-dependent peak shifts to higher binding energies for some tetrahedrite samples, and the amount of surface oxide species. In the tetrahedrite nanoparticles, the presence of Cu 2p doublets as well as the satellite Cu shake-up features indicate that Cu(II) is present, while the famatinite spectra lack these features. Analysis of the Cu Auger region and the Cu 2p region confirms for famatinite that all Cu species are Cu(I), but Cu(0) and Cu(I) could not be distinguished for tetrahedrite. Sb exists as Sb(III) in tetrahedrite and Sb(V) in famatinite based on the Sb 3d_3/2_ peak in famatinite being shifted to higher binding energies relative to the lower energy Sb 3d_3/2_ peak in the tetrahedrite spectra.^57,58^ Tetrahedrite spectra display evidence of oxide formation in the Cu 2p, Sb 3d, and S 2p regions. The intensity of the SO_*x*_ peak correlates both with the intensity of the Sb_*x*_O_*y*_ signal and the intensity of the Cu(II) peaks. Famatinite nanoparticles show signs of oxidation in the Sb 3d and S 2p regions, but peaks for both are notably less intense relative to tetrahedrite samples. Fits displayed in Figure S2a,b reveal an overall higher amount of oxygen species are present in tetrahedrite material, aligning with observations made in the Cu 2p, Sb 3d, and S 2p regions. Regarding the impact of dopants on the electronic structure of tetrahedrite and famatinite nanomaterials, shifts in the XPS spectra were only observed in select tetrahedrite samples in the Sb 3d and S 2p regions. Specifically, the Zn- and Fe-doped nanoparticle spectra were shifted in the Sb 3d region, while the Zn-, Fe-, and Co-doped spectra show a shift in the S 2p region, with all peak shifts being to higher binding energies. In contrast, no dopant dependent shifts were observed in any region of the famatinite spectra. This suggests that the electronic structure of famatinite is less sensitive to Cu-site doping than tetrahedrite, which is consistent with optical characterization that showed the band gap of doped tetrahedrite shifted from 1.88 to 2.04 eV while the band gap of doped-famatinite shifted from 0.87 to 0.95 eV.^26,27^

**Figure 4:**
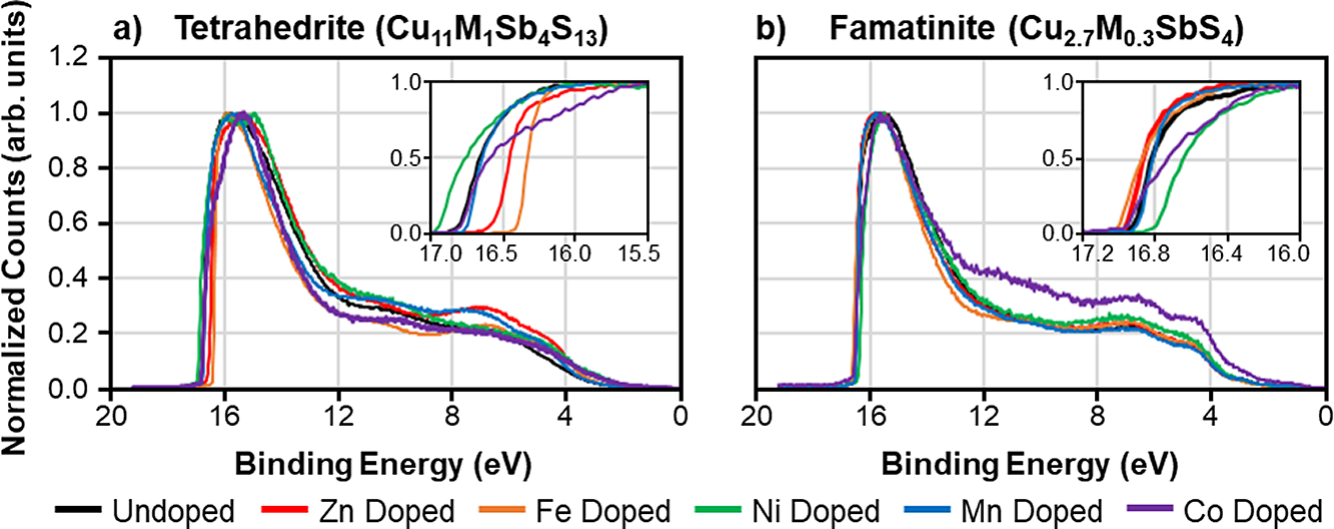
UPS spectra for (a) tetrahedrite (Cu_11_M_1_Sb_4_S_13,_ M = Zn, Fe, Ni, Mn, Co) nanoparticles and (b) famatinite (Cu_2.7_M_0.3_SbS_4_, M = Zn, Fe, Ni, Mn, Co) nanoparticles. The insets in (a) and (b) display the secondary electron cutoff of the tetrahedrite and famatinite nanomaterials, respectively. The legend identifies samples based on the identity of the dopant species (M), i.e., “Zn Doped” refers to the Cu_11_ZnSb_4_S_13_ tetrahedrite sample or the Cu_2.7_Zn_0.3_SbS_4_ famatinite sample.

Tetrahedrite and famatinite nanoparticles were characterized with UPS methods to examine valence structure and determine the impact of dopant incorporation on the work function of the materials. UPS results for tetrahedrite and famatinite nanoparticles are displayed in Figure 4a,b, respectively. All tetrahedrite and famatinite UPS spectra share a common spectral line shape, with the intense secondary electron cutoff at ∼14–16 eV and a lower intensity broad feature ranging from ∼12 eV to the Fermi level. The work function is calculated by subtracting the binding energy of the secondary electron cutoff from the energy of the He source (21.2 eV). A summary of the secondary electron cutoff, work functions, and linear fit data for all tetrahedrite and famatinite nanoparticles is available in Figures S3, S4, and Table S3. The undoped tetrahedrite nanoparticles possess a work function of 4.35 eV. Overall, the work function of the doped tetrahedrite nanoparticles ranges from 4.21 to 4.79 eV. For the famatinite nanoparticles, the undoped sample exhibits a work function of 4.67 eV, which is ∼0.3 eV higher than that of the undoped tetrahedrite sample. A smaller range in work function values from 4.57 to 4.77 eV is observed for the doped famatinite nanoparticles.

One study investigating nanoparticles synthesized by a hot injection method analyzed the UPS spectra of both tetrahedrite and famatinite, finding work functions of ∼4.7 and ∼4.8 eV, respectively.^28^ While the work function of the undoped famatinite nanomaterials in this study possessed a similar work function (4.67 eV), the polyol-synthesized undoped tetrahedrite nanoparticles displayed a lower work function (4.35 eV). Other studies obtained work functions ranging from 4.5 to 4.9 eV for undoped tetrahedrite nanoparticles synthesized by hot-injection.^37−39^ Only one study has previously characterized Cu-site doped tetrahedrite with UPS, obtaining work functions of 4.51–4.68 eV for the solid-state synthesized tetrahedrite.^36^ This study displayed less tunability in the work function relative to the polyol-synthesized Cu-site doped tetrahedrite. Additionally, dopant levels of these solid-state synthesized tetrahedrites were not consistent across all samples.^36^ For undoped famatinite nanoparticles and thin films, studies found work functions of 4.26^40^ and 4.61 eV, respectively.^41^ To this point, no studies have analyzed Cu-site doped famatinite with UPS methods. UPS data is heavily dependent on the surface composition of a material, so tetrahedrite and famatinite produced by other synthetic methods may possess ligand shells, impurity phases, or surface oxides that impact the work function. This potential difference in surface composition could explain the small differences between work function values derived in this work and other published studies.

### 3.3 EPR Spectroscopy

While XPS is a surface sensitive technique, EPR can detect surface and bulk paramagnetic species. Steady-state electron paramagnetic resonance spectroscopy (EPR) was performed on undoped and doped tetrahedrite and famatinite nanomaterials to identify paramagnetic centers and study how the local interatomic interactions of constituent and dopant species impact magnetic behavior. Both the copper and transition metal dopants each have at least one paramagnetic oxidation state, though there are additional considerations as to what may be detected. Cu(0) engaged in metallic bonding is diamagnetic, and will not be observed, but Cu(II) is readily observed. Transitions metals also generally have large zero-field splitting. As a result, high-spin non-Kramer’s ions, which have integer spin and, therefore, a nondegenerate ground spin state, cannot be observed by X-band EPR. This reduces the list of possible oxidation states of the dopants that may be detected directly to Mn(II), Fe(III), Co(II), Ni(III), and Zn(I).

**Figure 5:**
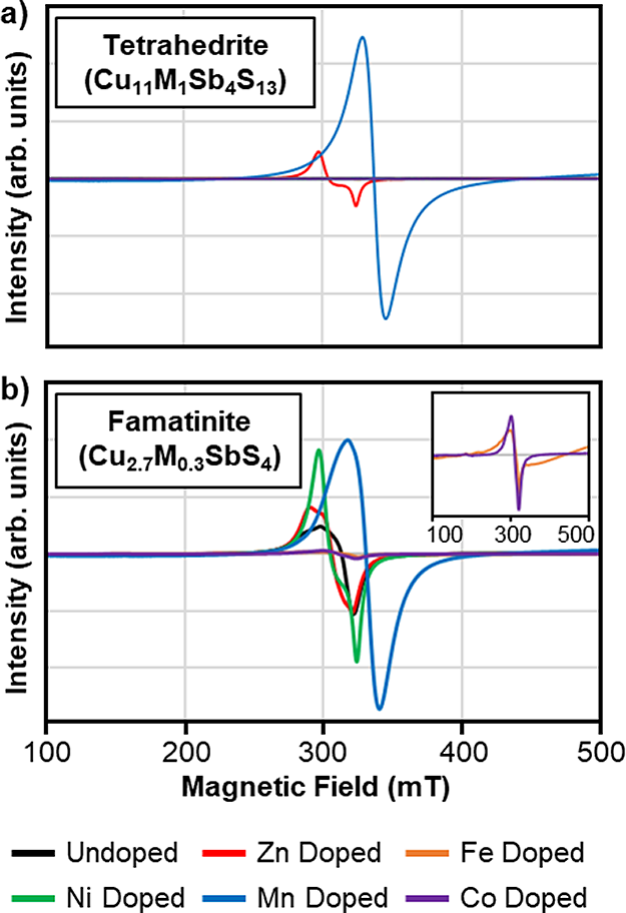
Electron paramagnetic resonance spectra for (a) tetrahedrite (Cu_11_M_1_Sb_4_S_13,_ M = Zn, Fe, Ni, Mn, Co) nanoparticles and (b) famatinite (Cu_2.7_M_0.3_SbS_4_, M = Zn, Fe, Ni, Mn, Co) nanoparticles. The inset in (b) displays a magnified view of the signal found for the Fe-doped and Co-doped famatinite samples. The Mn-doped signal in (a) is reduced by a factor of 3. The legend identifies samples based on the identity of the dopant species (M), i.e., “Zn Doped” refers to the Cu_11_ZnSb_4_S_13_ tetrahedrite sample or the Cu_2.7_Zn_0.3_SbS_4_ famatinite sample.

Examining the EPR spectra of transition metal-doped tetrahedrite complexes (Figure 5a) reveals that only the Mn-doped and Zn-doped samples exhibit an EPR signal. Individual EPR spectra for the Mn- and Zn-doped tetrahedrite samples are shown in Figure S5, with corresponding *g*-values listed in Table S4**.** The Mn-doped tetrahedrite nanoparticles (Figure S5a) exhibit an intense isotropic signal typical of Mn(II) with a *g*-value of 2.004.^62^ Hyperfine interactions (*I* = 5/2) are not resolved.^49,62^ There is no indication that Cu(II) is contributing to the spectral line shape in the Mn-doped tetrahedrite, although the intensity of the Mn(II) signal may be masking signals from other paramagnetic species. The signal for the Zn-doped sample shows asymmetry with *g*-values of 2.217 and 2.082, respectively (Figure S5b). The observation of paramagnetic Zn ions is rare and the expected *g*-value for Zn(I) is 1.99, which is inconsistent with the observed spectrum.^63^ The shape of EPR signal suggests the coordination environment of the paramagnetic species is axial with *g*_⊥_ > *g*_∥_, consistent with the paramagnetic Cu(II) centers being primarily found at the trigonal sites. A very similar spectral shape has been observed in the literature for dicopper coordination compounds and copper-containing solids experiencing spin exchange between nonidentical paramagnetic Cu(II) centers.^64,65^ Spin-exchange has also been reported in the EPR of tetrahedrite.^53^ Given the strong similarities of the spectral shape to literature reports of spin-exchanged copper systems, including copper sulfate pentahydrate and several copper coordination complexes, results suggest that the EPR signal in Zn-doped tetrahedrite arises from a spin exchange Cu(II)–Cu(II) system.^45,64,65^ It is possible that by doping with Zn (a diamagnetic species) the overall concentration of paramagnetic species in the sample is decreased, reducing the effects of relaxation such that a signal is observed.

The lack of an EPR signal for the polyol-synthesized undoped tetrahedrite nanoparticles described herein agrees with the work of Guler et al., who measured the EPR signature of undoped and Fe-doped tetrahedrites synthesized by a solid-state melting and recrystallization process.^52^ They reported that samples of undoped, solid-state synthesized tetrahedrite displayed no signal; however, their Fe-doped samples (Cu_11_Fe_1_Sb_4_S_13_) exhibited an intense EPR signal centered at a field value of ∼320 mT.^52^ Spin counting methodology was used to determine that their EPR signal stemmed from the formation of exchange-coupled pairs of Fe(III) and Cu(II), resulting in a composite paramagnetic center having a spin of *S* = 3/2.^52^ In contrast, the polyol-synthesized Fe-doped nanoparticles herein did not display a significant paramagnetic signal by EPR analysis. Furthermore, the XPS analysis suggested that Cu(II) was present in all polyol-synthesized tetrahedrite nanoparticles, but an associated EPR signature for the paramagnetic Cu species was surprisingly absent from the Fe-doped as well as from the undoped, Ni-doped, and Co-doped nanoparticle spectra. A literature report on the temperature dependence of the EPR signal in copper–antimony–sulfide materials showed increasing signal intensity with decreasing temperature, indicating significant effects of spin–lattice relaxation on the EPR signal.^54^ It is, therefore, possible that the EPR signal of Cu(II) in the tetrahedrites is being broadened to such an extent by relaxation effects that they are not observable at room temperature. Further exploration using temperature-dependent EPR methods could shed light on this possibility.

In contrast to the tetrahedrite, all famatinite nanomaterials display EPR signal (Figure 5b). Individual spectra can be found in Figure S6, and a summary of *g*-values is available in Table S4**.** Famatinite nanoparticles exhibit *g*-values between approximately 2.0–2.4. Since XPS analysis of the famatinite nanoparticles suggests there are no paramagnetic Cu species (such as Cu(II)) present, it is initially surprising that the undoped famatinite nanoparticles are EPR-active. However, XPS is a surface selective technique, whereas EPR spectroscopy will measure both surface and bulk paramagnetic centers. Alternatively, all famatinite nanomaterials may contain trace amounts of Cu(II) or other paramagnetic species at concentrations below the level of detection for XPS, but above the detection limit for EPR. A previous study published the room-temperature EPR spectra of undoped and Ni-doped famatinite nanoparticles, observing similar EPR line shapes as the undoped and Ni-doped famatinite nanoparticles herein.^53^ The only species in undoped famatinite that could give rise to a room temperature EPR signal at X-band is Cu(II), therefore, this signal is assigned accordingly. Similar to the study herein, EPR signal was detected despite XPS data showing only Cu(I) species were present in a study of CZTS nanomaterials at room temperature, leading authors to suggest that trace amounts of Cu(II) species (engendered by cationic disorder and nonstoichiometry) were present and responsible for the EPR signal.^50^

As detailed below, all EPR spectra measured for the undoped and doped famatinite samples show strong similarities in their *g*-factors with the exception of those doped with Fe and Mn. Based on the observations above, all EPR signals in famatinite samples are, therefore, assumed to originate from Cu(II), except in the case of the Fe- and Mn-doped compounds. The *g*-value for the Mn-doped famatinite nanoparticles is 2.039, markedly different from the other observed *g*-values for famatinites. It is greater than the *g*-value of 2.004 for the Mn-doped tetrahedrite nanoparticles (Figure S5a) and agrees with EPR signals for solid-state Mn(II) experiencing dipolar interactions,^51^ indicating a different magnetic environment or different magnetic interactions between the Mn and neighboring ions in famatinite as compared to tetrahedrite. This conclusion is further supported by a comparison of the line shape of the Mn-doped famatinite (Figure S5a) and tetrahedrite (Figure S6a). While the former appears entirely isotropic, the shape of the latter shows broadening on the low-field side of the spectrum. There is no discernible structure, so it is unclear whether this is due to hyperfine coupling of the electron spin to the copper nucleus (*I* = 5/2) or unresolved *g*-anisotropy. Given the lack of observable hyperfine interaction across all samples studied, it is more likely that the source is *g*-anisotropy, which indicates the environment at which the Mn-dopant is located in the crystal structure differs between tetrahedrite and famatinite.

The EPR spectrum of the Fe-doped famatinite nanoparticles (Figure S6d) also differs significantly from the others in that the signal is broadened and weak. The line shape agrees with the signal for Fe(III) found in Fe-doped ZnAl complexes, where the broadening is attributed to a high density of paramagnetic centers, leading to a significant contribution to the spectra from exchange interactions and dipolar couplings.^66^ Additionally, the spectral shape is consistent with the EPR measurements of Fe-doped tetrahedrites by Guler et al., where the observed signal was assigned to an Fe–Cu spin exchange pair; therefore, the signal of the Fe-doped sample studied herein is assigned as a spin exchange pair where the Fe-dopant is interacting with Cu(II), resulting in a composite spin system that is significantly broadened by the spin–spin interactions. The similarity between our spectrum and that of Guler et al. further strengthens the argument that spin-exchange plays a significant role in the magnetic interactions within this class of materials.^52^

The remainder of the EPR spectra of the undoped and doped famatinite nanoparticles (EPR spectra Figures 5 and S6; *g*-values Table S4), show very consistent *g*-factors indicating that the EPR signals arise from the same source–paramagnetic Cu(II) sites. All of the EPR spectra show clear signs of *g*-anisotropy, yet each spectrum is different, meaning the electronic environment in which the Cu(II) is located is structurally different and depends on the identity of the dopant. In the case of the undoped and Co-doped samples (Figure S6b,c), an additional feature is observed on the low field side of the spectrum, suggesting axial symmetry with *g*_∥_ > *g*_⊥_. This is consistent with Cu(II) in an environment with elongation on the unique axis of its coordination sphere. The Ni-doped sample (Figure S6e) differs from the others in that it has *g*_⊥_ > *g*_∥_, with *g*-values of 2.212 and 2.081, suggesting axial symmetry with compression, rather than elongation, along the *z*-axis, and it has a striking resemblance to the EPR spectra of Zn-doped tetrahedrite described above and other copper–copper spin exchange systems.^62,63^ Meanwhile, the Zn-doped famatinite (Figure S6f) has a line shape suggestive of a rhombic *g*-tensor, with *g* values at 2.323, 2.202, and 2.098.

The origin of the variations in the shape of the EPR spectra for samples with different dopants is unclear, but there are several possibilities. First, given the variation in size of the dopant ions, it is possible that the dopants are occupying different sites in the famatinite structure, and as a result, are altering the coordination environment around the paramagnetic Cu(II) sites. Alternatively, the dopants may be interacting electronically or magnetically with Cu(II) in unique ways altering the *g*-value of the observed spectrum. If the dopant ion and Cu(II) have significantly different *g*-values, a spin-exchange pair should appear at a location between that of the two spin-exchange partners. No significant shifts in *g*-value are evident in the data presented here with the exception of Mn-doped compounds, although spin–spin pairs of species with nearly identical *g*-values—like Cu–Cu spin pairs—cannot be identified this way. Alternatively, the observed complexity of the EPR signal could be the result of overlapping spectra of paramagnetic species at distinct sites–for example, surface versus bulk given the nanoscale size of the materials. Finally, these nanomaterials are polycrystalline, which may lead to different electronic environments for the Cu(II) giving rise to different signals. Identifying distinct contributions to the EPR spectrum from heterogeneous samples requires carefully designed studies. Given the complexity and variety of possible sources for the observed EPR spectra reported above, determining the exact origin requires further investigations.

### 3.4 Cumulative Comparison of XPS, UPS, and EPR Data

Results from the XPS and UPS analysis of polyol-synthesized tetrahedrite materials revealed some small dopant dependent shifts while corresponding EPR characterization provided insight into coordination environments. Analysis of the Cu 2p region reveals the presence of Cu(I), Cu(II), and possible Cu(0) in tetrahedrite. It is anticipated that Cu_12_Sb_4_S_13_ contains two Cu(II) and ten Cu(I) with possible Cu enrichment up to the composition of Cu_14_Sb_4_S_13_, decreasing the amount of Cu(II). The oxidation state of Sb in tetrahedrite was determined to be Sb(III). In the Cu 2p, Sb 3d, and S 2p regions, certain peaks suggest the formation of surface oxide compounds with Cu, Sb, and S. The incorporation of Zn and Fe dopants was observed to shift the XPS spectra to higher binding energies in both the Sb 3d and S 2p regions, and incorporating Co shifts the spectra to higher binding energies in the S 2p region. Tetrahedrite nanoparticles exhibited dopant-influenced changes to the material work function, which ranged from 4.21–4.79 eV. While XPS data suggested most samples contained Cu(II), an EPR signal was only observed for the tetrahedrite nanoparticles doped with Zn and Mn. The lack of signal could be attributed to spin–lattice relaxation as well as spin–spin coupling of Cu(II) centers, which is more significant for materials with high concentrations of paramagnetic species. Both of which would result in significant broadening of the Cu(II) signal to a point where no signal is observed.

The XPS, UPS, and EPR spectra of the famatinite nanoparticles contain distinct differences relative to the tetrahedrite spectra, with the higher sensitivity of the EPR characterization relative to XPS revealing additional information. In the Cu 2p region, 2p_3/2_ and 2p_1/2_ peaks consistent with Cu(0) or Cu(I) species were observed while the features that signify the presence of Cu(II) were not present. The Sb 3d_3/2_ peak in famatinite was shifted to higher binding energies relative to the tetrahedrite spectra, which revealed the presence of Sb(V) in famatinite instead of the Sb(III) found in tetrahedrite. In the S 2p region, the S 2p_3/2_ and 2p_1/2_ peaks were shifted to higher binding energies relative to tetrahedrite as observed in the Sb 3d region. Generally, signs of surface oxidation were absent from the Cu 2p regions of all famatinite XPS spectra, but evidence of surface oxidation was observed in the Sb 3d and S 2p regions, albeit at a lower level than in tetrahedrite. None of the famatinite nanomaterials display dopant-dependent shifts in the XPS spectra, and a smaller range of work functions (4.57–4.77 eV), relative to tetrahedrite, were observed for the famatinite nanomaterials. In contrast to tetrahedrite, all famatinite nanoparticles surprisingly displayed EPR signal, which is attributed to the presence of trace amounts of paramagnetic Cu(II) in the famatinite nanoparticles at levels below the detection limit of XPS and/or the presence of paramagnetic manganese and iron for those respective doped compounds. Furthermore, EPR results suggested that the transition-metal dopant species experience varied local ion coordination environments, interacting differently with the paramagnetic Cu(II) sites as evidenced by changes in EPR line shape.

## 4 Conclusions

Tetrahedrite (Cu_12_M_1_Sb_4_S_13_, M = Cu, Zn, Fe, Ni, Mn, or Co) and famatinite (Cu_2.7_M_0.3_SbS_4_, M = Cu, Zn, Fe, Ni, Mn, or Co) nanoparticles synthesized by a modified polyol process were confirmed to be single-phase by XRD with elemental compositions within an acceptable range as determined by EDS. For this extensive sample set, the electronic properties of and magnetic interactions within the nanoparticles were evaluated by complementary XPS, UPS, and EPR techniques. Overall for the XPS and UPS data, it was found that tetrahedrite was more impacted by the incorporation of Cu-site dopants than famatinite. It is significant to note that the tetrahedrite samples were shown by XPS to contain Cu(II), which must be present for charge-balance in Cu_12_Sb_4_S_13_ and also may be due to surface oxidation. Yet most of those samples (with the exception of Zn- and Mn-doped) did not show an EPR signal, which is attributed to significant spin–spin or spin–lattice relaxation. In contrast, XPS characterization of all famatinite samples did not indicate the presence of Cu(II), however an EPR signal was observed. EPR detects the bulk and the surface with a lower detection limit than XPS, revealing the presence of some oxidized copper, in the form of Cu(II), is indeed present in famatinite. A significant outcome of this research study is showing the importance of using the complementary characterization techniques of photoelectron spectroscopy and electron paramagnetic resonance in concert to fully elucidate the oxidation state of metals (Cu in particular) in solid-state nanomaterials.

By tuning electronic and magnetic properties, promising ternary copper chalcogenide materials such as tetrahedrite and famatinite can be optimized for thermoelectric and photovoltaic applications. To further investigate the electronic structure of tetrahedrite and famatinite, one could increase the dopant level or utilize other synthetic-based approaches for controlling the composition of the material. Additionally, XPS analysis reveals surface oxidation, particularly for the tetrahedrite materials. As a result, future projects will study approaches to inhibit or induce surface oxidation. To further understand the magnetic behavior of doped tetrahedrite and famatinite nanomaterials, temperature-dependent changes to the EPR spectra of the materials could be studied. In particular, this would nullify signal broadening from spin–lattice relaxation, potentially allowing for the observation of Cu(II) signals. Other studies could look to further understand the different environments that dopant atoms appear to occupy in famatinite. Increasing knowledge of the electronic and magnetic properties of tetrahedrite and famatinite materials are important steps toward designing efficient, cost-effective, and earth abundant green energy materials.

## Supporting Information Available

Crystallographic data derived from Rietveld refinements, Cu Auger XPS signals for all samples, representative peak fitting in the Sb 3d region of the XPS spectra, UPS data for all nanoparticle samples, individual EPR plots with associated *g*-values, and *g*-values from EPR characterization of the Cu–Sb–S nanomaterials (PDF)
