# Supplementary material for: Investigating Cu-Site Doped Cu–Sb–S Nanoparticles Using Photoelectron and Electron Paramagnetic Resonance Spectroscopy
Source: J Phys Chem C Nanomater Interfaces. 2024 Aug 8;128(33):13888–99. doi: 10.1021/acs.jpcc.4c02602 (PMC11345821; doi:10.1021/acs.jpcc.4c02602)
Supplement: Supplementary file 1 — jp4c02602_si_001.pdf [file jp4c02602_si_001.pdf]

Supporting Information: **Investigating Cu-Site Doped Cu-Sb-S Nanoparticles Using Photoelectron and Electron Paramagnetic Resonance Spectroscopy**

Authors:
Jacob E. Daniel†, S. Ivan Weaver†, Brad R. Matthias†, River Golden†, Gavin M. George†, Christian Kerpal^, Carrie L. Donley‡, Lauren E. Jarocha†, Mary E. Anderson†*

†Department of Chemistry, Furman University, Greenville SC 29613
‡Chapel Hill Analytical and Nanofabrication Lab, Department of Chemistry, University of North Carolina, Chapel Hill, NC 27599
^Department of Physics and Astronomy, UNC Asheville, Asheville, NC 28804
*Corresponding Author: maryelizabeth.anderson@furman.edu

Included:

Table S1: Rietveld refinement calculations for tetrahedrite ($Cu_{11}M_1Sb_4S_{13}$, M = Cu, Zn, Fe, Ni, Mn, Co) nanoparticles, including lattice parameter *a*, grain size, and Chi^2 values. (Page 2)

Table S2: Rietveld refinement calculations for famatinite ($Cu_{2.7}M_{0.3}Sb_4S_{13}$, M = Cu, Zn, Fe, Ni, Mn, Co) nanoparticles, including lattice parameters *a* and *c*, grain size, and Chi^2 values. (Page 2)

Figure S1: Cu Auger XPS signals for tetrahedrite and famatinite nanoparticles. (Page 3)

Figure S2: Representative peak fitting in the Sb 3d region of the XPS spectra for tetrahedrite and famatinite nanoparticles. (Page 4)

Peak Fitting Procedure and Discussion (Page 5)

Figure S3: UPS graphs of the secondary electron cutoff fit for tetrahedrite nanoparticles (Page 6)

Figure S4: UPS graphs of the secondary electron cutoff fit for famatinite nanoparticles (Page 7)

Table S3: Summary of UPS data for tetrahedrite and famatinite nanoparticles including equation of the linear fit, secondary electron cutoff, and estimated work function. (Page 8)

Figure S5: Individual EPR plots with associated g-values for the Mn- and Zn-doped tetrahedrite nanoparticles. (Page 9)

Figure S6: Individual EPR plots and associated g-values for famatinite nanoparticles. (Page 10)

Table S4: Calculated g-values for all tetrahedrite and famatinite nanoparticles that display an EPR signal. (Page 11)

Table S1: Rietveld refinement of XRD data for tetrahedrite nanoparticles.

Sample	a (Å)	Grain Size (Å)	Chi^2 ‡
$Cu_{12}Sb_4S_{13}$	10.340 (1)	166 (2)	1.0074
$Cu_{11}ZnSb_4S_{13}$	10.361 (2)	166 (3)	1.5030
$Cu_{11}FeSb_4S_{13}$	10.351 (1)	188 (2)	1.6869
$Cu_{11}NiSb_4S_{13}$	10.330 (1)	241 (1)	1.0848
$Cu_{11}MnSb_4S_{13}$	10.400 (2)	164 (1)	0.9775
$Cu_{11}CoSb_4S_{13}$	10.3407 (8)	197 (3)	1.0363

‡The Chi^2 value is a goodness-of-fit measurement for the Rietveld refinement performed by the PDXL2 software.

Table S2: Rietveld refinement of XRD data for famatinite nanoparticles.

Sample	a (Å)	c (Å)	Grain Size (Å)	Chi^2 ‡
Cu_3SbS_4	5.382 (2)	10.752 (6)	58.6 (5)	1.2149
$Cu_{2.7}Zn_{0.3}SbS_4$	5.372 (3)	10.687 (9)	49.5 (3)	1.0491
$Cu_{2.7}Fe_{0.3}SbS_4$	5.380 (1)	10.743 (4)	82.5 (6)	1.2043
$Cu_{2.7}Ni_{0.3}SbS_4$	5.385 (2)	10.725(7)	60.8 (3)	1.5587
$Cu_{2.7}Mn_{0.3}SbS_4$	5.384 (2)	10.723 (6)	78.8 (3)	1.0938
$Cu_{2.7}Co_{0.3}SbS_4$	5.3834 (1)	10.753 (4)	97.5 (6)	1.5128

‡The Chi^2 value is a goodness-of-fit measurement for the Rietveld refinement performed by the PDXL2 software.

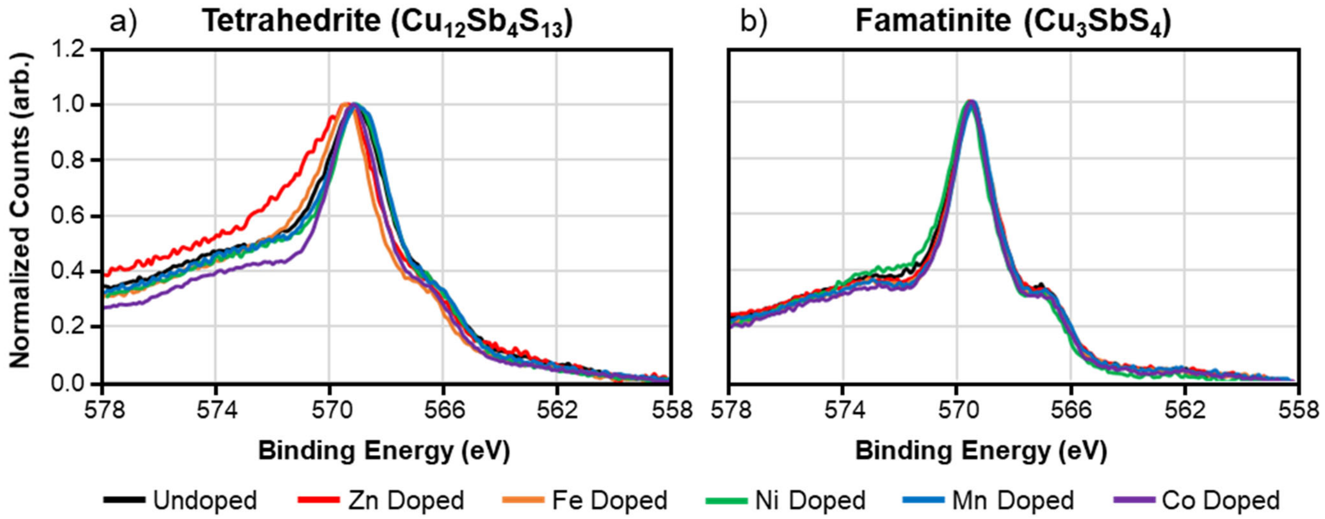

Figure S1: Cu Auger region of the XPS spectra for a) tetrahedrite ($Cu_{11}M_1Sb_4S_{13}$, *M*=Cu, Zn, Fe, Ni, Mn, or Co) and b) famatinite ($Cu_{2.7}M_{0.3}SbS_4$, *M*=Cu, Zn, Fe, Ni, Mn, or Co) nanoparticles.

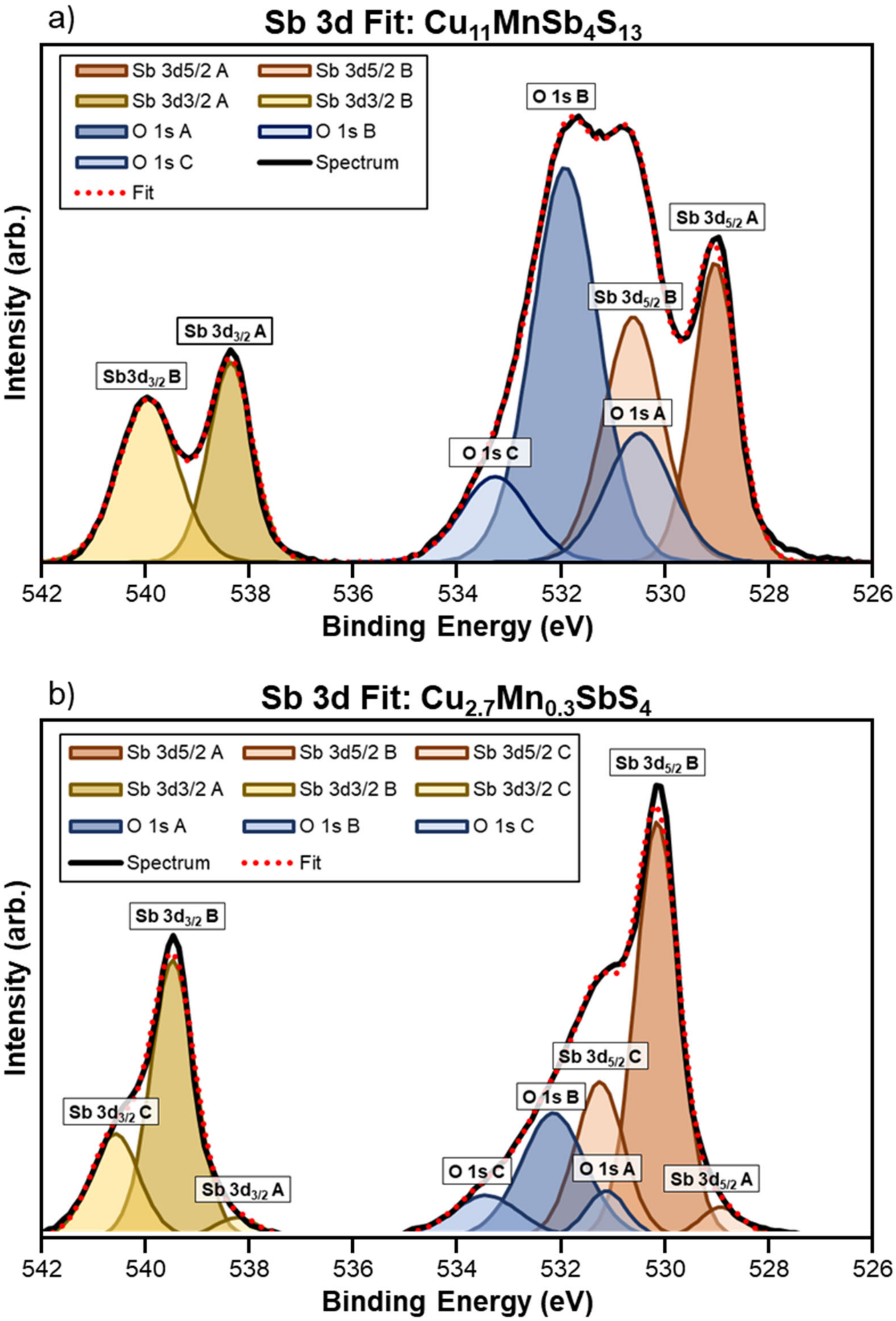

Figure S2: Representative peak fits in the Sb 3d region from a) the Mn-doped tetrahedrite sample ($Cu_{11}MnSb_4S_{13}$) and b) the Mn-doped famatinite sample ($Cu_{2.7}Mn_{0.3}SbS_4$) showing the overlap between the Sb $3d_{5/2}$ and O 1s signals.

Peak Fitting Procedure and Discussion

Peak fitting for the Sb 3d and O 1s region was done with the Kratos ESCApe software. Shirley baselines were used, and the peak shapes were Gauss*Lorentz with blend=0.3. The Sb 3d peaks were fit first using the Sb 3d 3/2 region as a guide since there was no overlap with the O 1s peaks. The peak splitting was 9.34eV for the two peaks in the Sb 3d doublet, the two peaks in the doublet had the same FWHM, and the area ratio between them was 0.667. Once a reasonable fit was obtained for the Sb 3d 3/2 region of the spectrum, then additional peaks were added corresponding to the O 1s contribution.

While oxygen is not a stoichiometric component of these samples, it was observed in all of them. Evidence of oxidized sulfur is observed in both compounds; and probably oxidized copper and antimony in the tetrahedrite samples. Typically, the O 1s signal from metal oxides appear at lower binding energies than the corresponding metal hydroxides, and both are potentially present in these samples. In addition, higher binding energy O 1s peaks (in the range of 533-534 eV) are often attributed to tightly bound surface water. This research did not attempt to try to assign the O 1s peak fits because there are so many potential sources of oxygen, and there is likely considerable overlap within the O 1s envelope. For this research, the peak fitting was primarily to distinguish the Sb 3d signal from the O 1s signal for quantification purposes.

An additional note regarding the peak position of the dominant O 1s peak (labeled O 1s B in Figure S2), is that the binding energies of this peak for the tetrahedrite samples ranged from 531.07-531.74 eV and for famatinite samples ranged from 531.89-532.33 eV.

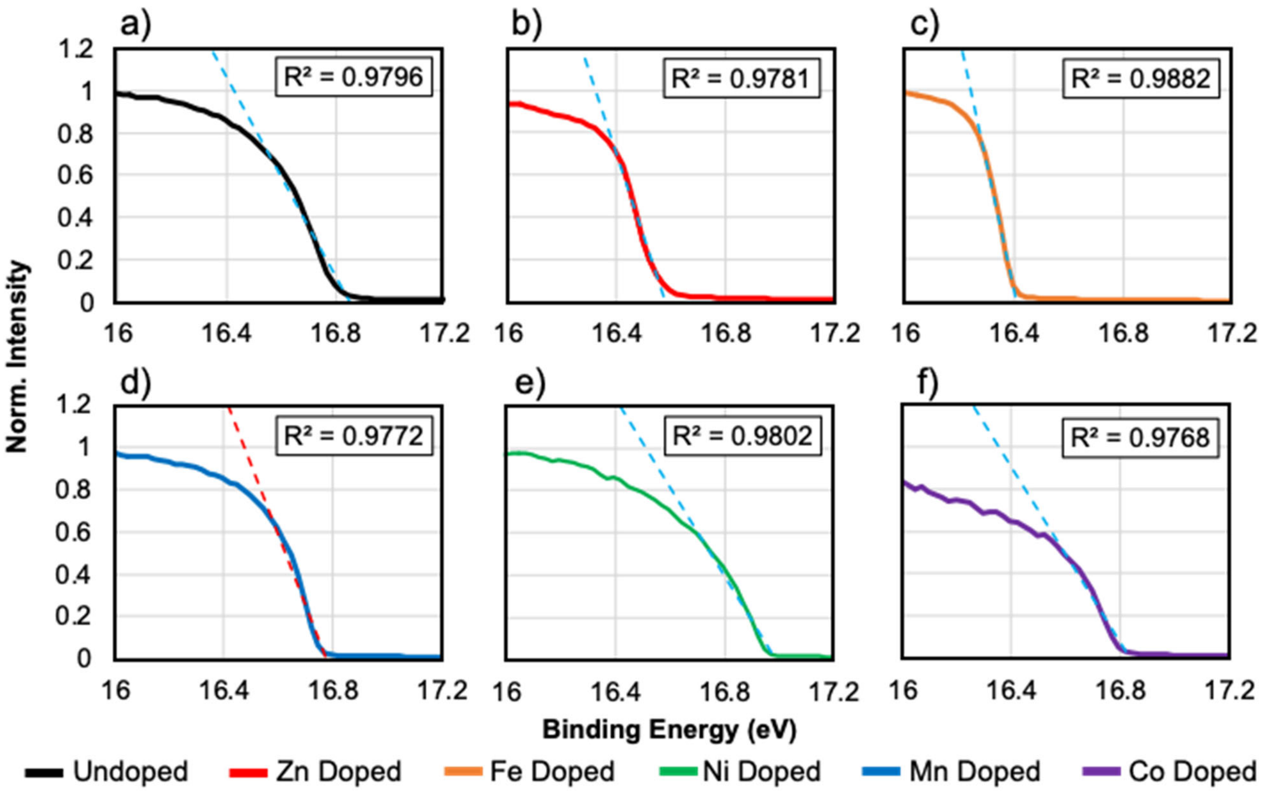

Figure S3: Position of the secondary electron cutoff for the (a) undoped, (b) Zn-doped, (c) Fe-doped, (d) Ni-doped, (e) Mn-doped, and (f) cobalt-doped tetrahedrite ($Cu_{11}M_1Sb_4S_{13,}$ M = Zn, Fe, Ni, Mn, Co) nanoparticles. Magnitude of the secondary electron cutoff was calculated by finding the x-intercept of a linear regression applied to the higher binding energy edge of the UPS spectra. R^2 value for all linear fits are included in the inset, and the equation of the line for each fit are listed in **Table S3**.

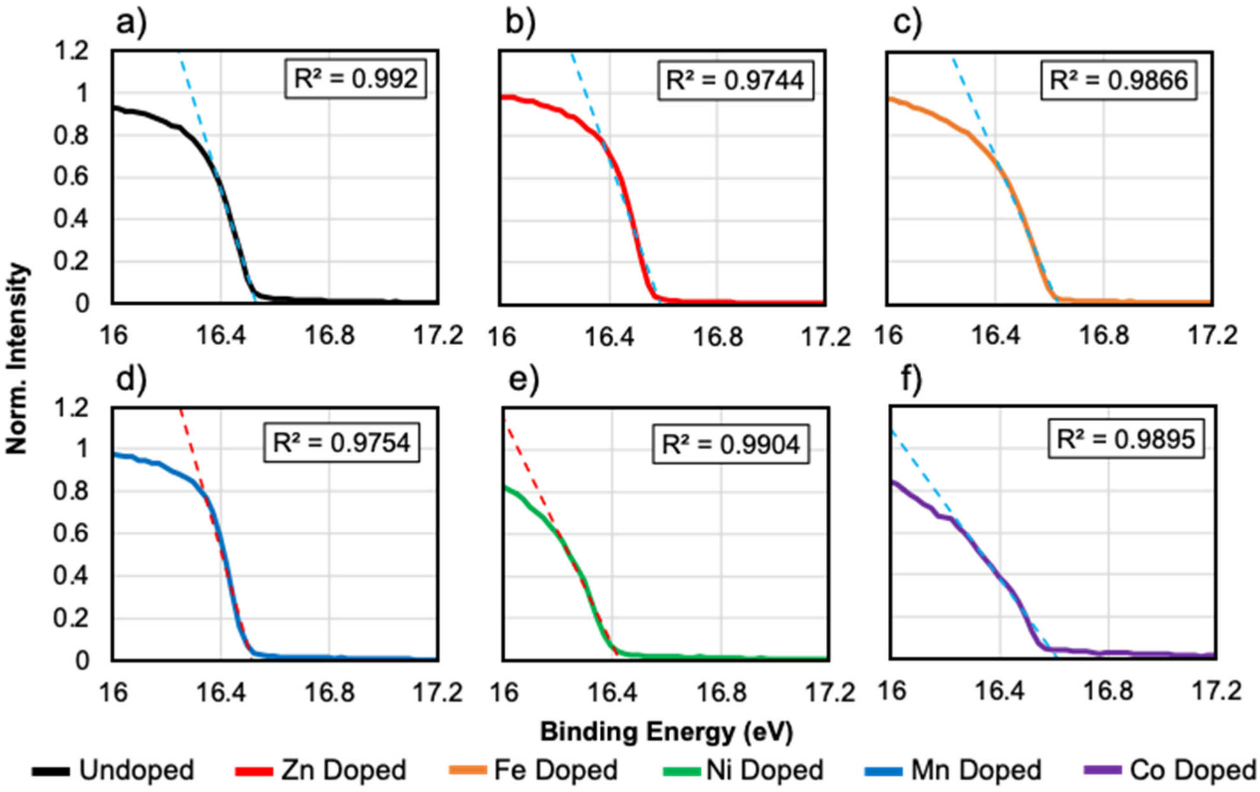

Figure S4: Position of the secondary electron cutoff for the (a) undoped, (b) Zn-doped, (c) Fe-doped, (d) Ni-doped, (e) Mn-doped, and (f) cobalt-doped famatinite ($Cu_{2.7}M_{0.3}SbS_4$, M = Zn, Fe, Ni, Mn, Co) nanoparticles. Magnitude of the secondary electron cutoff was calculated by finding the x-intercept of a linear regression applied to the higher binding energy edge of the UPS spectra. R^2 value for all linear fits are included in the inset, and the equation of the line for each fit are listed in **Table S3.**

Table S3: UPS data summary for tetrahedrite and famatinite nanoparticles

Tetrahedrite Target	Linear Fit[a]	R^2	SEC (eV)[b]	Est. Φ (eV)[c]
$Cu_{12}Sb_4S_{13}$	y = -2.357x + 39.721	0.9796	16.85	4.35
$Cu_{11}ZnSb_4S_{13}$	y = -3.896x + 64.598	0.9781	16.58	4.62
$Cu_{11}FeSb_4S_{13}$	y = -5.951x + 97.641	0.9882	16.41	4.79
$Cu_{11}NiSb_4S_{13}$	y = -2.102x + 35.711	0.9772	16.99	4.21
$Cu_{11}MnSb_4S_{13}$	y = -3.324x + 55.767	0.9802	16.78	4.42
$Cu_{11}CoSb_4S_{13}$	y = -2.078x + 34.974	0.9768	16.83	4.37
**Famatinite Target**	**Linear Fit[a]**	**R^2**	**SEC (eV)[b]**	**Est. Φ (eV)[c]**
Cu_3SbS_4	y = -4.158x + 65.727	0.9920	16.53	4.67
$Cu_{2.7}Zn_{0.3}SbS_4$	y = -3.596x + 59.663	0.9744	16.59	4.61
$Cu_{2.7}Fe_{0.3}SbS_4$	y = -2.997x + 49.856	0.9866	16.63	4.57
$Cu_{2.7}Ni_{0.3}SbS_4$	y = -4.525x + 49.856	0.9754	16.41	4.79
$Cu_{2.7}Mn_{0.3}SbS_4$	y = -2.698x + 44.312	0.9904	16.43	4.77
$Cu_{2.7}Co_{0.3}SbS_4$	y = -1.765x + 29.321	0.9895	16.61	4.59

[a]Linear fit extrapolated from high binding energy edge of UPS spectra **(Fig. 4).**
[b]Secondary electron cutoff (SEC) determined by calculating x-intercept of linear fit for high binding energy edge of UPS spectra.
[c]Work function (ϕ) calculated by subtracting SEC from the energy of the He I ionization source (21.2 eV).

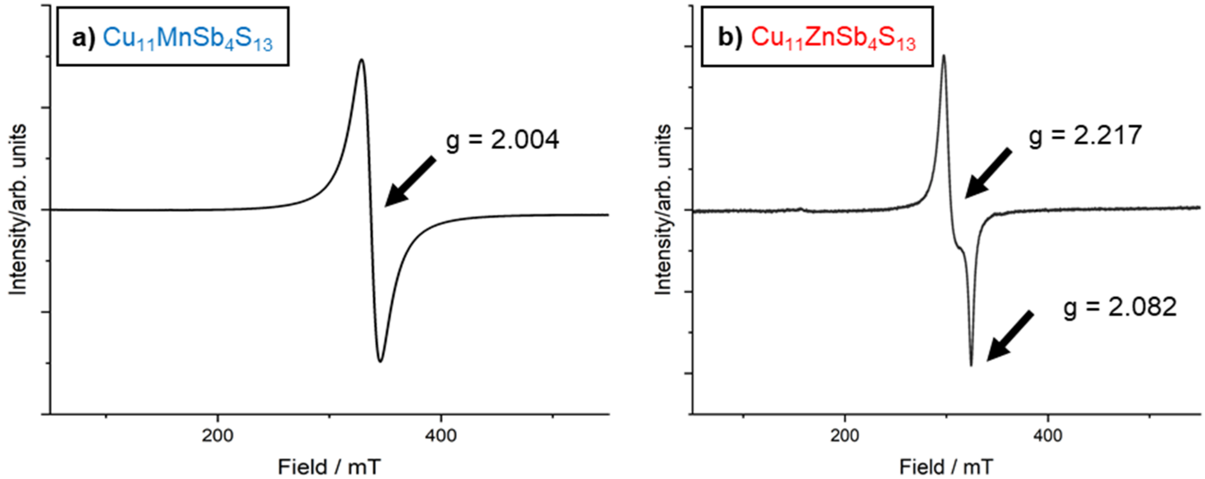

Figure S5: Individual EPR spectra labelled with the calculated g-values for the a) $Cu_{11}MnSb_4S_{13}$ and b) $Cu_{11}ZnSb_4S_{13}$ tetrahedrite nanoparticles.

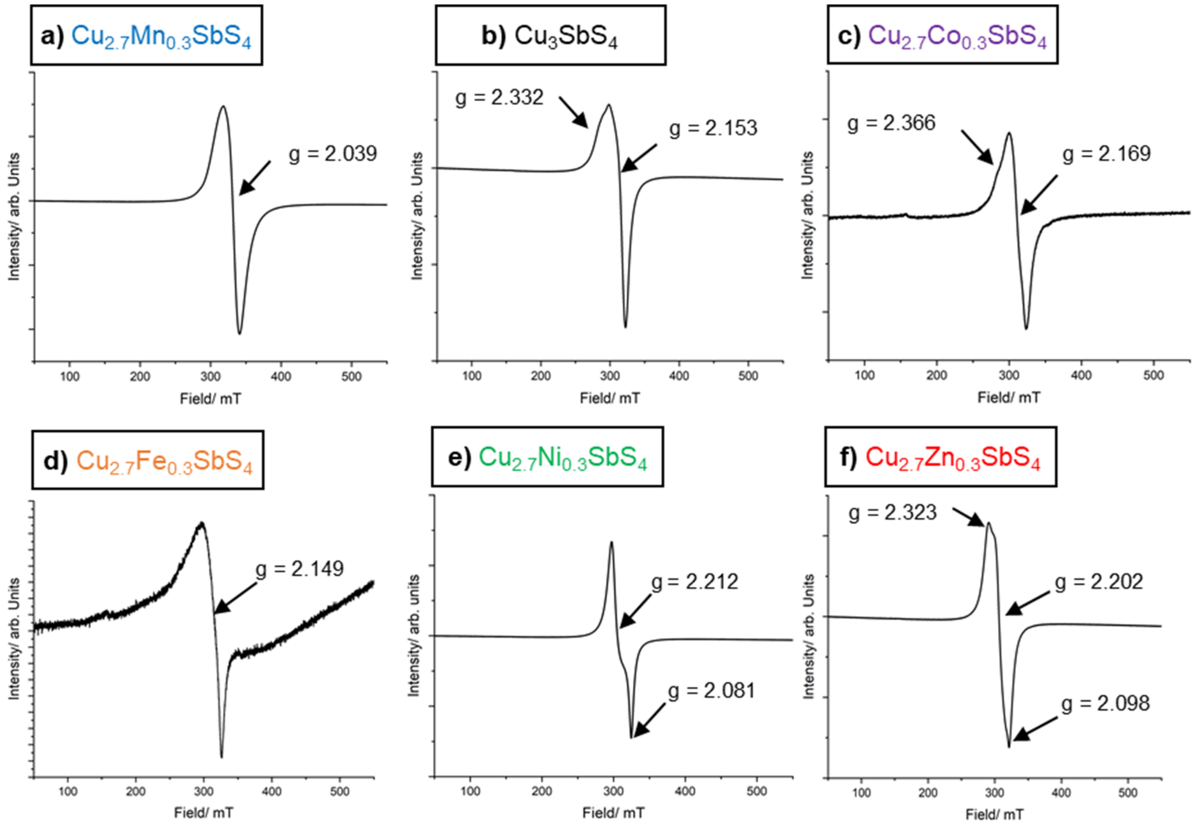

Figure S6: Individual EPR spectra labelled with the calculated g-values for the a) $Cu_{2.7}Mn_{0.3}SbS_4$, b) Cu_3SbS_4, c) $Cu_{2.7}Co_{0.3}SbS_4$, d) $Cu_{2.7}Fe_{0.3}SbS_4$, e) $Cu_{2.7}Ni_{0.3}SbS_4$, and f) $Cu_{2.7}Zn_{0.3}SbS_4$ famatinite nanoparticles.

Table S4: Calculated g-values for tetrahedrite and famatinite nanoparticles

Sample	g_1	g_2	g_3
$Cu_{11}MnSb_4S_{13}$	2.004	-	-
$Cu_{11}ZnSb_4S_{13}$	2.082	2.217	-
$Cu_{2.7}Mn_{0.3}SbS_4$	2.039	-	-
Cu_3SbS_4	2.153	2.332	-
$Cu_{2.7}Co_{0.3}SbS_4$	2.169	2.366	-
$Cu_{2.7}Fe_{0.3}SbS_4$	2.149	-	-
$Cu_{2.7}Ni_{0.3}SbS_4$	2.081	2.212	-
$Cu_{2.7}Zn_{0.3}SbS_4$	2.098	2.202	2.323
